# Neuromuscular exercise in children with Down Syndrome: a systematic review

**DOI:** 10.1038/s41598-022-19086-8

**Published:** 2022-09-02

**Authors:** Eliana-Isabel Rodríguez-Grande, Olga-Cecilia Vargas-Pinilla, Martha-Rocio Torres-Narvaez, Nelcy Rodríguez-Malagón

**Affiliations:** 1 Doctoral Program in Clinical Epidemiology, Pontificia Universidad Javeriana, Bogotá, Colombia; 2 School of Medicine and Health Sciences, GI Rehabilitation Sciences, Universidad del Rosario, Bogotá, Cundinamarca Colombia; 3 Department of Clinical Epidemiology and Biostatistics, Pontificia Universidad Javeriana, Bogotá, Colombia

**Keywords:** Health care, Health occupations

## Abstract

The effects and the prescription parameters of therapeutic exercise are not clear. For this reason, is needed to determine the effect of neuromuscular exercise on balance, muscle strength and flexibility specifying the parameters and characteristics of effective interventions in children between 6 and 12 years and adolescent between 13 and 18 years with Down Syndrome. The present study is a systematic review of effectiveness outcomes balance, muscle strength and flexibility in this population. The databases of PubMed, PEDro, EMBASE, SCIELO, Lilacs, Cochrane library were searched from May to December 2021. We recruited randomized controlled trials (RCTs) which met the inclusion criteria in our study. Ten studies were included. The interventions included mechanotherapy, vibration, and use of different unstable surfaces. The exercise frequency ranged from 3 to 5 days a week, and the duration of each session was between six and 15 min. The frequency was between two and three times a week for 6 and 12 weeks and the intensity were between 60 and 80% of maximal voluntary contraction. Neuromuscular exercise in different modes of application was associated with increases in chest and lower limb muscle strength mean 8.51, CI [2.35–14.67] kg and (21.54 [1.64, 41.43]) kg. Balance also improved when the mode of application was isokinetic training and core stability exercises (− 0.20 [− 0.29, − 0.12]) evaluated with stability index. Neuromuscular exercise appears to be effective for the improvement of both lower limb and chest muscle strength and balance in children over 8 years. No evidence was found in children under 8 years.

## Introduction

Children with Down Syndrome (DS) between 6 and 12 years and adolescent between 13 and 18 years exhibit delayed motor development compared with typically developing children^1^. DS involves alterations in balance, strength, and muscular endurance, which affect postural control and generate atypical motor development in addition to cognitive disability. Approximately 10% of children with DS can sit in an upright position and exhibit an independent walking pattern under 3 years, and approximately 95% exhibit these abilities between 3 and 6 years^2^.

In general, children with DS retain the typical sequence of motor development, but basic motor skills such as walking, running, jumping, climbing, throwing and catching are attained late in childhood^3^. The hypotonia and muscle weakness interfere with intermuscular coordination and processing of proprioceptive information affecting the postural control, functionality, and quality of life of the child. For example, the impairments in postural control in this population may constrain the performance of activities of daily living and potentially impact other aspects of functioning^4^. Previous studies have reported postural control deficits in individuals with DS^5^, including a systematic review with focus in the interactions among the components of the International Classification of Functioning (ICF)^4^.

These characteristics restrict independence in mobility, which increases the demand for monitoring and support by caregivers, a situation that becomes more evident in the adolescent stage because of the new relationships established with friends and the school environment in which the child interacts^6,7^.

To promote mobility and independence in daily activities, children and adolescents with DS can participate in therapeutic physiotherapy interventions that aid the development of motor skills and improve functional performance with therapeutic exercise, among other intervention alternatives^8^. Therapeutic exercise involves the application of physical exercise parameters such as intensity, frequency, and duration for therapeutic purposes. Therapeutic exercise categories include aerobic, anaerobic, resistance, neuromotor and neuromuscular exercise^9^.

The neuromuscular exercise and neuromotor exercise modalities can be similar, they are used in different training scenarios, some of which are therapeutic and some of which are focused on increasing performance in athletes^9^. Neuromuscular exercise aims to improve sensorimotor control (i.e., the ability to produce controlled movement through coordinated muscle activity) and to facilitate neuromuscular control. Thus, neuromuscular exercise seeks to improve the unconscious response of muscles to signals related to dynamic joint stability, which is the ability of the joint to remain stability during movement execution. To achieve this goal, neuromuscular exercise improves variables such as muscle strength, flexibility, and balance^10,11^.

Neuromuscular exercise activates neurophysiological processes of intermuscular coordination that, together with increased muscle strength and postural responses, contribute to stability in the execution of motor patterns within the functional repertoire of children and adolescents with DS. There are different ways to perform neuromuscular exercise, including the use of unstable surfaces such as balls or mats, mechanotherapy equipment, isokinetic techniques, and bodyweight resistance exercises^12–15^.

To the best of our knowledge, no systematic reviews have addressed the effects of neuromuscular exercise in the pediatric population with DS. In addition, the neuromuscular exercise prescription parameters that are effective in improving the outcomes proposed in this review are unknown. This is a very important gap in knowledge given that the set of prescription parameters configure a dose of exercise that must be sufficient to achieve the proposed therapeutic objectives, if there is no clarity in the parameters or is no therapeutic window, it is possible that therapeutic interventions are not effective^16,17^.

There are several primary studies that have examined the effects of neuromuscular interventions in children and adolescents with DS. Although some published reviews on the effects of physical therapy interventions have included neuromuscular exercise in their analysis, the inclusion of recent primary studies may improve the certainty of the evidence^8,12^ and facilitate decision-making in rehabilitation.

Previous systematic reviews of evidence regarding muscle exercise have included randomized controlled trials (RCTs) and quasi-experimental studies, making comparisons between studies despite the differences in their methodological quality. In addition, previous systematic reviews have examined different research questions, different populations, and different therapeutic interventions^8,18,19^.

The main aim of the current study was to synthesize the existing research evidence on the effects of neuromuscular exercise. The secondary aim was to determine the parameters and characteristics of effective interventions to improve balance, muscle strength and flexibility in children and adolescents with DS between 4 and 18 years of age. This synthesis of the best available evidence may help to improve the prescription of these interventions in therapeutic approaches for this population and facilitate the development of motor skills and functional performance in children with DS.

## Methods

We adhered to the Preferred Reporting Items for Systematic Reviews and MetaAnalyses (PRISMA) guidelines^20^. This analysis was prospectively registered on Open Science Framework (OSF) and it is available in https://doi.org/10.17605/OSF.IO/JM2RB. Ethical and internal review board approval was not required because no human or animal subjects were involved.

### Eligibility criteria

#### Design

A systematic review of the literature was conducted to identify RCTs.

#### Type of participants

Children with DS between 4 and 18 years.

#### Type of interventions

All neuromuscular interventions of therapeutic exercise with specific prescription parameters in terms of intensity, frequency, duration, among others were included.

#### Outcomes

For inclusion, studies identified in the literature search that included results for at least one of the outcomes included in the review: muscle strength, balance, and flexibility.

### Exclusion criteria

Texts not available in full text: study authors were contacted to provide full text. If no response was obtained, the study was excluded.

### Search and identification of studies

Search terms were generated from the Population, Intervention, Comparison and Outcome (PICO) components of the following question: ¿What is the effect of neuromuscular therapeutic exercise on strength, balance, and flexibility in children with DS? These terms were adapted according to the different databases explored. A systematic search was conducted in the Pubmed, EMBASE, SCIELO, Lilacs, Cochrane Library and Epistemonikos databases^21–25^. In addition, other sources of evidence were consulted to allow the identification and analysis of published or unpublished literature (gray literature) that had not been detected by the systematic search. This process was carried out through manual searches in reference lists of documents found in the review of databases. This process was developed during the months of May to December 2021.

We use Mesh terms^26^ for searches in English and DeCS terms^27^ for searches in Spanish. In addition, we include free terms and the combination of the previous ones for the construction of the algorithms in each of the databases. The terms used were: down syndrome'/exp, Down syndrome, mongolism, trisomy ('infancy'/exp OR 'infancy') 'child'/exp, therapeutic exercise/exp, exercise, neuromuscular, exercise, resistance training, physical, activity, therapeutic, resistance, training, stretching, balance, rehab* OR kinesiotherap*.

### Selection of the studies

The final selection of the studies was made independently by two reviewers who were both physiotherapists, one with training as an epidemiologist and the other with a Master’s degree in Exercise and Sports Rehabilitation (MRTN and OCVP). The reviewers checked all titles and abstracts and excluded those that were considered irrelevant to the review because they did not meet the eligibility criteria of RCT design and inclusion of at least one of the prioritized outcomes.

Subsequently, the reviewers reviewed the full text of the studies verifying the eligibility criteria. Each reviewer generated Bib Tex files of the studies they considered eligible, and identified duplicates were eliminated using a bibliographic manager. The choice of each study was determined by consensus after independent review by the two reviewers. In cases where there was no consensus, a third reviewer decided on eligibility.

### Extraction and management of variables

All variables considered relevant for the comparison of the studies and measurement of outcomes were extracted. Data on the type, mode, frequency, intensity, duration of the interventions, location in which the interventions were performed (e.g., outpatient clinic, home) and person in charge of applying the intervention (e.g., physical therapist, other professional, family member or caregiver) were extracted from predesigned forms.

For the population, data were obtained regarding age, sex, sample size of each group and cognitive engagement.

Data were extracted for muscle strength, balance, and flexibility outcomes. For balance, data were obtained on center of mass displacement or time to maintain a balanced posture. For muscle strength, data were reported in pounds, kilograms of force, or Newtons. For flexibility, data on muscle elongation were obtained.

### Evaluation of study quality

Two independent reviewers (MRTN and OCVP) assessed the risk of bias of each included study using the Cochrane Collaboration’s risk of bias assessment tool^28^. They rated each study as having a “low risk of bias,” “high risk of bias,” or “unclear risk of bias,” taking into account six domains: random sequence generation (selection bias), allocation masking (selection bias), blinding of participants and personnel (performance bias), blinding of outcome assessment (detection bias), incomplete outcome data, and selective reporting (reporting bias). The risk of bias rating was analyzed using Revman 5.1 software.

Disagreements in bias assessment were resolved by a third-party evaluator (EIRG).

### Synthesis of data

The selected body of evidence was organized by prioritized outcomes. Within each outcome, we described the characteristics of the population, the parameters of the interventions including the mode of exercise applied, the frequency, intensity and duration of the interventions in these studies, and the quantitative results achieved with their level of significance. This information is presented in Table 1.

**Table 1:** Characteristics of the included studies.

Reference	Participants	Interventions	Outcomes, measuring instrument	Application parameters: Intensity, frequency, duration, therapeutic exercise mode	Results resented here are the final measurements for each group and p value
Shield N, 2013	Number of participants: 68 Boys: 38 Girls: 30 Age: 17.9 ± 2.6 years Severity of intellectual disability: Mild–moderate	Group 1: PRT Group 2: Social program	Muscle strength: 1 MR	Type: Anaerobic Mode: Gym machines Frequency: 2 days a week for 10 weeks Duration: 45–60 min Intensity: 60%–80% of 1 MR, 3 sets of 12 repetitions Intervention applied by: Physiotherapy student	Chest press (kg) 1 MR, Group 1: after week 11: 43.6 ± 16.0; after week 24: 42.7 ± 18.4 Chest press (kg) 1 MR, Group 2: after week 11: 32.0 ± 11.7; after week 24: 35.0 ± 14.0 Leg press (kg) 1 RM Group 1: after week 11: 128.1 ± 46.8; after week 24: 133.3 ± 59.5 Leg press (kg) 1 MR Group 2: after week 11: 92.4 ± 49.9; after week 24: 101.3 ± 48.3 The PRT group increased their upper and Lower limb strength at week 11 compared To the control group. Only their lower limb Muscle strength at week 24
Shield N, 2010	Number of participants: 23 Boys: 17 Girls: 6 Age: 15. ± 1.6 years Severity of intellectual disability: Mild: 6 children; moderate: 15 children; severe: two children	Group 1: PRT Group 2: Usual recreational and leisure activities	Muscle strength: 1 MR	Type: Anaerobic Mode: Gym machines Frequency: 2 days a week for 10 weeks Duration: 45–60 min Intensity: 60%–80% of 1 MR, 3 sets of 12 repetitions Intervention applied by: Physiotherapy student	Chest press (kg): Group 1: after week 10: 55 ± 24 Chest press (kg) Group 2: after week 10: 44 ± 12 Leg press (kg): Group 1: after week 10: 132 ± 50 Leg press 1 MR (kg) Group 2: after week 10: 97 ± 43 PRT group improve in lower limb muscle strength compared to the control group (MD 36 kg, 95% CI 15 to 58)
Eid MA, 2017	Number of participants: 31 Boys: 17 Girls: 14 Age: 10.26 ± 0.79 years Severity of intellectual disability: Mild	Group 1: Conventional physiotherapy and isokinetic training Group 2: Conventional physiotherapy	Muscle strength: Isokinetic dynamometer (maximum peak torque) Balance: Biodex platform	Type: Mode: Isokinetic Machines and Balance Training Frequency: 3 days a week for 12 weeks Duration: 60 min Intensity: Speeds 90–120°/sec Intervention applied by: Physiotherapists	Maximum peak torque (Nm) Group 1: Right knee flexors: post: 29.06 ± 2.46 group 2: post: 27 ± 2.47 p: 0.02; Right knee extensors: Group 1 post: 45.06 ± 2.86, Group 2: post 42 ± 3.46, p: 0.01; Left knee flexors: Group 1 post: 25.23 ± 3.28, group 2: 22.18 ± 1.88 p:0.003; Left knee extensors: Group 1 post: 40.73 ± 3.03, Group 2 post: 37.93 ± 3.47 p: 0.02 Group 1: Anteroposterior stability index: post: 1.19 ± 0.18; Group 2: post: 1.34 ± 0.09, p:0.008 Mediolateral stability index: Group 1: post: 1.23 ± 0.08; Group 2: post: 1.54 ± 0.13 p: 0.0001 Each group showed significant improvements in postural balance and peak torque of knee flexors and extensors (P < 0.05), with significantly greater improvements observed in the study group compared with the control group (P < 0.05)
Jankowicz A, 2012	Number of participants: 40 Boys: 20 Girls: 20 Age: 16.8 years Severity of Intellectual disability: Mild	Group 1: Balance training Group 2: No exercise	Static one-legged balance. Using Duo Balance. Centre Of Gravity (COG) trajectory length and time frame in which the vertical projection of COG remained within the 13 mm radius circle	Type: Mode: Unstable surfaces (ball and foam) Frequency: 2 days a week for 12 weeks Duration: 45 min Intensity: NR Intervention applied by: NR	COG trajectory length (mm) Group 1: OA, post: 1615.62 ± 694.6; OC, post: 2591.46 ± 1166.6 COG trajectory length (mm) Group 2: OA, post: 1871.19 ± 1531.8; OC, post: 3107.5 ± 2216.0 Percentage of time (seconds) circle radius 13 mm: Group 1: OA, post: 68.02 ± 15.5; OC post: 44.02 ± 20.19 Percentage of time frame (seconds): Group 2: OA, post: 60.01 ± 23.78; OC, post: 40.7 ± 22.1
Lin H, 2012	Number of participants: 92 Boys: 46 Girls: 46 Age: G1 15.6 ± 3.6 years Age: G2 14.9 ± 3.9 years Severity of Intellectual disability: Mild	Group 1: exercise program based on virtual reality with Wii, treadmill Group 2: No exercise Group 1: Training to ride a bike without training wheels Group 2: No activity	Muscle strength: Manual dynamometer (lbs)	Type: Mode: Unstable surfaces (ball and foam) Frequency: 3 days a week for 6 weeks Duration: 35 min Intensity: 50%–65% fc max. Intervention applied by: Senior occupational therapist	Muscle strength (lbs): Group 1 hip flexors: 17.33 ± 2.15; group 2: 16.20 ± 1.97 p: 0.01 Hip extensors: Group 1:14.07 ± 1.24, Group 2: 13.02 ± 2.04, p:0.01 Hip abductors: Group 1: 14.46 ± 1.73; Group 2: 13.37 ± 1.82, p:0.004 Knee flexors: Group1: 16.27 ± 1.81; Group 2: 15.02 ± 1.45 p: 0.02 Knee extensors: Group 1: 15.75 ± 1.94; Group 2: 14.65 ± 1.23, p: 0.03 Plantar flexors: Group 1: 14.04 ± 1.28; Group 2: 13.30 ± 1.46, p: 0.01
Ulrich D, 2011	Number of participants: 46 Boys: 20 Girls: 26 Age: G1 12 ± 1.9 years Age: G2 12.4 ± 2.2 years Severity of intellectual disability: Mild	Group 1: Bicycle intervention Group 2: no intervention	Isometric muscle strength: Manual dynamometer (kg) Unipedal static equilibrium time (seconds)	Type: Mode: Bicycle Frequency: 5 days Duration: 75 min Intensity: NR Intervention applied by: Volunteers from a foundation	Main effects of group were found for knee flexion in the right and left legs. The group 1 had significantly greater knee flexion in the right leg ( P:0.041) and left leg (P:0.026) overall than the group 2
Villaroya MA, 2013	Number of participants: 57 Boys: 37 Girls: 20 G1 Age: 15.93 ± 2.48 G2 Age: 15.64 ± 2.93 Severity of Intellectual disability : NR	Group 1: Vibration training Group 2: No exercise	Static balance. Displacement of pressure centers at the beginning and end of the study on the pressure platform. Eyes open fixed surface (C1); eyes closed fixed surface (C2); eyes open unstable surface (C3); eyes closed unstable surface (C4)	Type: Mode: Vertical vibration platform Power Plate Pro 5 Frequency: 3 days a week for 20 weeks Duration: 18 min Intensity: 28-Hz frequency, 28-mm amplitude, and 2.2-g vibration dose Intervention applied by: Investigator	No significant p-values. They report the difference, not the values of the measured outcomes
Eid MA, 2015	Number of participants: 30 Boys: 17 Girls: 13 Age: G1: 8.93 ± 0.7 years Age: G2: 9.26 ± 0.79 years Severity of intellectual disability: Mild	Group 1: Conventional physiotherapy and vibration Group 2: Conventional physiotherapy	Bipedal balance and gait training with muscle stretching Balance platform Manual dynamometer (lbs)	Type: Mode: Bipedal postures and gaits Frequency: 3 days a week for 24 weeks Duration: 60 min physiotherapy + 18 min vibration Intensity: Vibration amplitude of 2 mm and frequency of 25 to 30 Hz Intervention applied by: Physiotherapist	Muscle strength (lbs): Group 1: Knee flexors: 15.65 ± 1.78; Group 2: 14.3 ± 1.66, p: 0.04 Knee extensors: Group 1:16.04 ± 1.6; Group 2: 14.76 ± 0.91, p: 0.01 Balance Indices (average): Group 1: ML: 1.09 ± 0.15; Group 2: 1.24 ± 0.09, p: 0.001 AP: Group 1: 0.92 ± 0.8; Group 2: 1.05 ± 0.08, p: 0.0001 Overall: Group 1:1.19 ± 0.14; Group 2: 1.37 ± 0.12, p: 0.004
Emara, 2016	Number of participants: 30 Boys: 17 Girls: 13 Age: G1 10.88 + \-0.52 years Age: G2 10.93 + \-0.58 years Severity of Intellectual disability: NR	Group 1: Exercise and vibration Group 2: Exercise	Manual dynamometer (N) Balance and gait training	Type: Mode: Power plate, rolls, wedges, and mats Frequency: 3 days a week for 12 weeks Duration: 60 min physiotherapy + 9 min vibration Intensity: Vibration amplitude 2–6 mm, frequency 25 to 30 Hz Intervention applied by: Physiotherapist	There was significant increase in lower limb strength in both groups when compared with their pre- and post-treatment results. Comparison of post-treatment results revealed more increase in lower limb strength in the Group 1
Aly S, 2016	Number of participants: 30 Boys: 21 Girls: 9 Age: G1 8.11 ± 1.26 years Age: G2 8.34 ± 1.07 years Severity of intellectual disability: Mild–moderate	Group 1: Conventional physiotherapy and core stability exercises Group 2: Conventional Physiotherapy	Biodex balance system: Circular platform—multiaxial apparatus and Jeffrey stability exercises	Type: Mode: Mat and Swiss balls Frequency: 3 days a week for 8 weeks Duration: 45–60 min Intensity: NR Intervention applied by: NR	Group 1: Anteroposterior stability index: Group 1: 0; Group 2: 1.8 ± 0.3, p: 0.0001 ML: Group 1: 1.21 ± 0.25, group 2: 1.62 ± 0.4. 0.002 Overall: Group 1: 1.74 ± 0.37, Group 2: 2.42 ± 0.49, p: 0.0001

Group 1, Intervention; Group 2, Control group; MR, Maximum Resistance; pre, before-intervention values; post, after-intervention values; NR, no reported, ML, mediolateral, PRT, Progressive resistance training.

Meta-analysis was performed when similarities were found in the components of the PICO question in at least two studies, as well as in the instruments used for the measurement of the outcomes and the qualification of the risk of bias. When the measurement instruments were different, the data were converted to common units.

For meta-analyses, data on population characteristics, randomization methods, outcome measures, duration of follow-up and methods of analysis were extracted from each study in a database previously designed in Excel. Direct comparisons made between the intervention and a control group defined as educational activities, recreational activities, or continuity with activities of daily living or another intervention of interest for this review were considered.

For the prioritized outcomes, averages and standard deviations were extracted from the available data. Standardized differences from the mean (SMD) and 95% confidence intervals (95% CI) were calculated to allow comparability of data. Heterogeneity between trials was assessed using the chi-square test, a statistical significance level of *p* < 0.05 and the value of statistic I^2^. When the data exhibited heterogeneity with an I^2^ greater than 70%, the results were combined using the random effects model and the 95% CI was calculated^29,30^. All the analysis above was performed using Revman 5 software^31^.

### Evaluation of the certainty of the evidence

Evaluation of the certainty of the evidence found was carried out using the GRADE approach^32^. In this approach, the evaluation of evidence goes beyond the evaluation of the methodological quality, because the evidence found for each outcome was graded considering *the risk of bias, inconsistency, directness or indirectness of the evidence and imprecision, risk of selective publication of outcomes, and dose–response gradient*. The first four criteria were rated using a three-level ordinal scale: *very serious, serious and not serious*. The classification options for risk of selective publication of outcomes were: *not detected, strong suspicion*. The classification options for effect size were*: no, large, very large*. The classification options for presence of confounding factors were: *no, will reduce the demonstrated effect, suggests spurious effect*. The classification options for dose–response gradient were: *no, yes, no*^33^.

## Results

### Study selection

**Figure 1:**
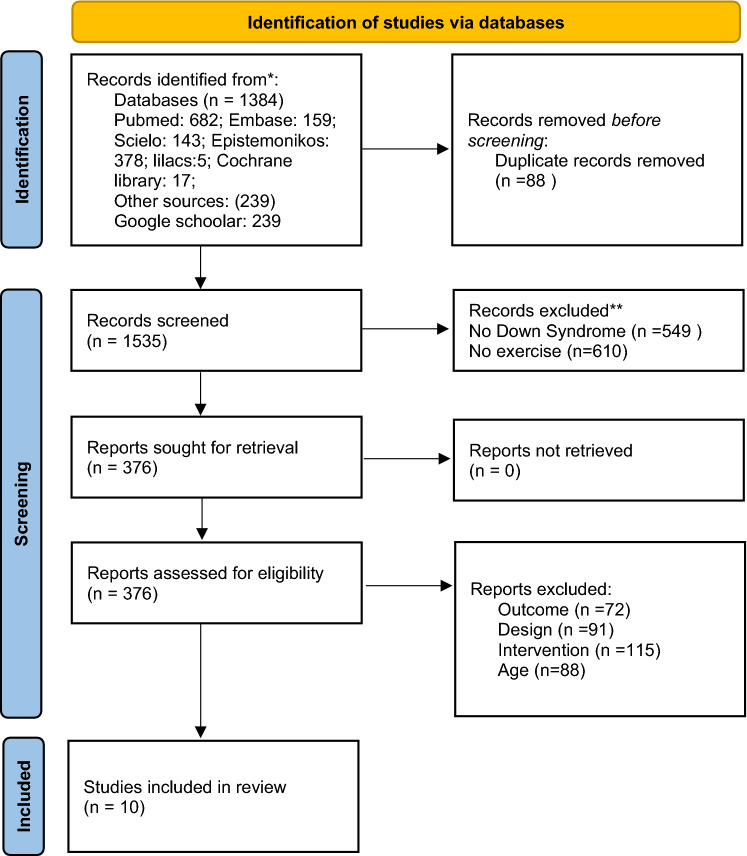
Flow chart of studies included. Adapted from: Page MJ, McKenzie JE, Bossuyt PM, Boutron I, Hoffmann TC, Mulrow CD, et al. The PRISMA 2020 stat.

A total of 1384 studies were identified in the systematic literature search. From other sources, including the bibliographic references of the studies found in the systematic search, 239 additional studies were identified, resulting in a total of 1623 identified studies. Of these studies, 88 were excluded because of duplication and 1159 were excluded based on reviewing the titles and abstracts. In total, 376 studies were reviewed in full text by two reviewers, of which 366 were excluded for not meeting the eligibility criteria. Finally, 10 primary studies were included. Figure 1 shows a flow chart of studies identified and included in the body of evidence.

## Assessment of the risk of bias of included studies

### Allocation (selection bias)

Six studies^14,15,34–37^ presented a low risk of bias because they reported masking of randomization, whereas four studies^38–41^ presented a high risk of bias because they did not report the methods by which participants were assigned, and the personnel in charge of maintaining random assignment were not masked.

### Blinding

Because of the nature of the interventions used, the risk of bias assessment of each study considered the blinding of the outcome assessors in each study. Three studies^35,38,40^ presented a high risk of bias, while seven^14,15,34,36,37,39,41^ presented a low risk of bias.

### Outcomes with incomplete data

Three studies presented a high risk of bias because they reported incomplete data by not including data from participants who did not achieve the expected results^40^ or did not indicate the number of participants included in the reported results^36,39^. The remaining studies reported all data from the study sample.

### Selective report

Eight studies presented a low risk of selective reporting because they included all the outcomes that were measured; one study had high risk^39^ and one more had unclear risk of bias^36^.

**Figure 2:**
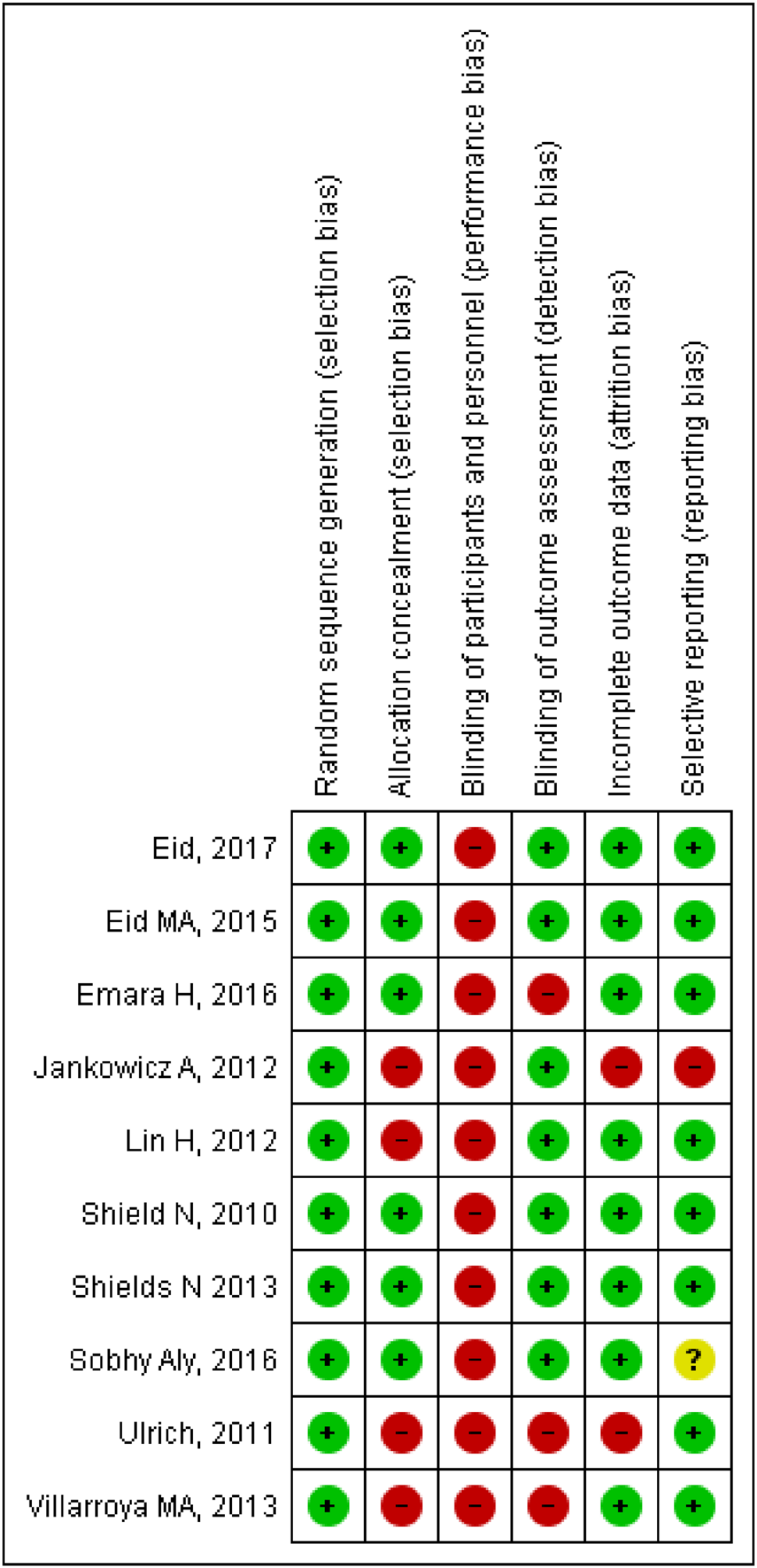
Risk of bias summary: review authors’ judgments about each risk of bias item for each included study. Revman 5. https://training.cochrane.org/online-learning/core-software-cochrane-reviews/revman.

**Figure 3:**
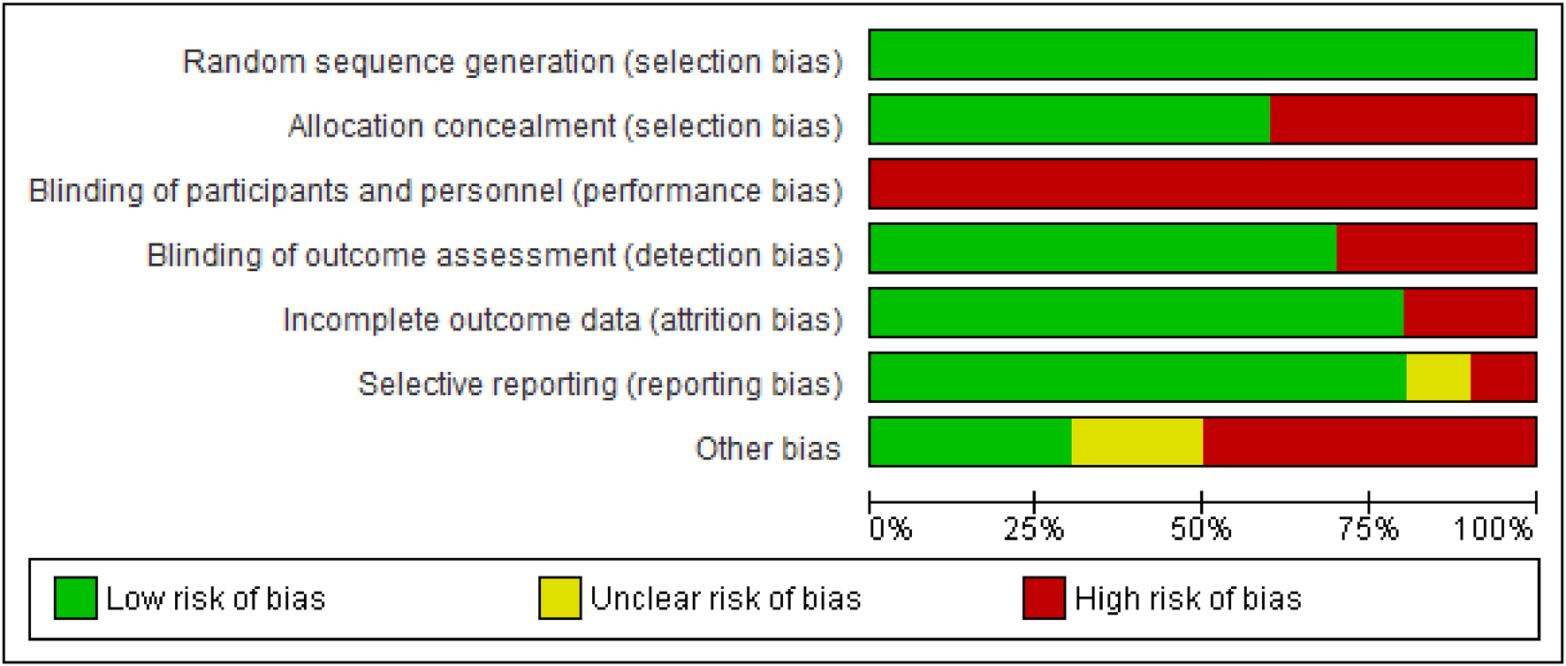
Risk of bias graph: review authors’ judgments about each risk of bias item presented as percentages across all included studies. Revman 5. https://training.cochrane.org/online-learning/core-software-cochrane-reviews/revman.

This information is summarized in Figs. 2 and 3.

### Modes of application of neuromuscular exercise in physiotherapy interventions in children aged 4 to 18 years

Two studies involved the use of mechanotherapy equipment available in gym for muscle strength training^14,34^. Three studies applied therapeutic vibration^15,35,38^, which was combined with conventional physiotherapy interventions in two studies^15,35^. Two studies used different unstable surfaces for balance training^36,39^, and one study used Nintendo Wii and virtual reality^41^. One study used isokinetic exercise^37^ and another used bicycle training^40^.

### Frequency, intensity and duration of neuromuscular exercise

In the two studies that examined muscle strengthening exercises using mechanotherapy equipment, the frequency was twice a week for 10 weeks^14,34^. In the studies that applied vibration^15,35,38^, the frequency was between three times per week for 24 weeks^15^ and three sessions every month for 20 weeks^38^ to 12 weeks^35^.

Exercise using unstable surfaces was applied with a frequency between two and three times a week for 8 and 12 weeks and a volume of three sets of 12 repetitions for each muscle group that was exercised^36,39^.

Lin et al.^41^ applied exercise three times a week for 6 weeks, whereas Eid et al.^37^ applied a frequency of three times a week for 6 months. The duration of the sessions varied between 35 and 75 min and the intensity, a parameter mentioned in few of the selected studies, was between 60 and 80% of maximal voluntary contraction in the studies that included exercise using mechanotherapy equipment^14,34^. Lin et al. ^41^ used the speed and inclination of the treadmill as intensity criteria, which started at 2.0 kph (0% inclination) and ended with an average speed of 3.0 kph (58° elevation).

### Effectiveness of neuromuscular exercise on the outcomes included in the review

No evidence was found for the outcome of flexibility in this population. Table 1 shows the characteristics of the studies included in this review.

### Muscle strength

Seven studies evaluated chest and lower limb muscle strength primarily^14,15,34,35,37,40,41^. Neuromuscular exercise in the different modes of application presented in this review was associated with significant increases in chest (8.51 [2.35,14.67] kg) and lower limb (21.54 [1.64, 41.43] kg) muscle strength (Table 2 and Appendix 1). The certainty of the evidence for the outcome of muscle strength was between moderate and high, Table 2.

**Table 2:** Evaluation of the certainty of the evidence presented for each outcome.

Certainty evaluation							No. of patients		Effect		Certainty	Importance
No. of studies	Study design	Risk of bias	Inconsistency	Indirect evidence	Imprecision	Other considerations	Neuromuscular Exercise	Control	Relative (95% CI)	Absolute (95% CI)		
**Chest muscle strength. Intervention: Mechanotherapy. Measuring instrument: Maximum resistance (MR) (Shield 2010 and 2013) (Follow-up: Mean 11 weeks; evaluated using MR (kg))**												
2	Randomized trials	Not serious	Not serious	Not serious	Serious^a^	None	45	46	–	MD 8.51 higher. (2.35 to 14.67)	⨁⨁⨁◯ MODERATE	CRITICAL
**Muscle strength of the lower limbs. Intervention: Mechanotherapy. Measuring instrument: Maximum resistance (MR) (Shield 2010 and 2013) (Follow-up: Mean 10 weeks; evaluated using MR (kg))**												
2	Randomized trials	Not serious	Not serious	Not serious	Serious^a^	None	45	46	–	MD 21.54 higher. (1.64 to 41.43)	⨁⨁⨁◯ MODERATE	CRITICAL
**Muscle strength of the lower limbs. Intervention: Isokinetic training. Instrument: Maximum peak torque (Newtons) (Eid 2017) (Follow-up: 12 weeks; evaluated using Maximum peak torque (Newtons))**												
1	Randomized trials	Not serious	Not serious	Not serious	Not serious	None	16	15	–	MD 2.68 higher (1.68 to 3.68)	⨁⨁⨁⨁ HIGH	CRITICAL
**Hip and knee muscle strength. Intervention: Exercise with treadmill and Wii. Instrument: Manual dynamometry (Lbs) (Lin 2012) (Follow-up: 18 weeks)**												
1	Randomized trials	Not serious	Not serious	Not serious	Not serious	None	46	46	–	MD 1.08 higher. (0.8 higher to 1.36 higher)	⨁⨁⨁⨁ HIGH	CRITICAL
**Knee muscle strength. Intervention: Isometric training. Instrument: Manual dynamometer (kg) (Ulrich 2011) (Follow-up: weekly for 12 months)**												
1	Randomized trials	Very serious^b^	Not serious	Not serious	Not serious	None	19	27	–	MD 3.18 higher. (1.87 higher to 4.5 higher)	⨁⨁◯◯ LOW	CRITICAL
**Knee muscle strength. Intervention: Conventional physiotherapy and therapeutic vibration (Eid 2015 and Emara 2016) Instrument: Manual dynamometry (Newtons) (Follow-up: 12 weeks)**												
2	Randomized trials	Serious	Not serious	Not serious	Serious^c^	None	32	30	–	MD 2.53 higher. (1.89 higher to 3.16 higher)	⨁⨁◯◯ LOW	CRITICAL
**Balance. Intervention: Conventional physiotherapy plus isokinetic training/core stability exercises (Eid 2017 and Sobhy 2016). Instrument: Stability Index (Biodex System)) (Follow-up: 12 weeks)**												
2	Randomized trials	Not serious	Not serious	Not serious	Not serious	None	16	15	–	MD 0.2 lower (0.29 lower to 0.12 lower)	⨁⨁⨁⨁ HIGH	CRITICAL
**Balance. Intervention: Neuromuscular exercise using unstable surfaces and balloons (Jankowics 2012). Instrument: Path length center of gravity (mm) (Follow-up: 12 weeks)**												
1	Randomized trials	Very serious^d^	Not serious	Not serious	Very serious^e^	None	20	20	–	MD 336.54 lower (948.52 lower to 275.44 higher)	⨁◯◯◯ VERY LOW	CRITICAL
**Unipedal balance. Intervention: Isometric training and unipedal balance (Ulrich 2011). Instrument: Unipedal balance maintained (seconds) (Follow-up: 12 months)**												
1	Randomized trials	Very serious^d^	Not serious	Not serious	Serious	None	17	27	–	MD 2.54 higher. (0.62 higher to 4.45 higher)	⨁◯◯◯ VERY LOW	CRITICAL

CI, Confidence Interval; MD, Mean Difference.

Explanations.

^a^Very wide confidence intervals. In one of the studies, the change is not statistically significant.

^b^The trend is high risk 5/ 7.

^c^Large confidence intervals.

^d^High risk 5/7.

^e^Very large confidence intervals.

### Balance

Seven studies evaluated balance^15,35–40^. Interventions with neuromuscular exercise were associated with significant improvement in balance when their mode of application was isokinetic training and core stability exercises (− 0.20 [− 0.29, − 0.12]) (Table 2 and Appendix 1).The certainty of evidence for balance was highly variable, ranging from very low to high, mainly because of the imprecision of the results obtained in the primary studies. The information is presented in Table 2.

## Discussion

The present review included articles reporting neuromuscular exercise with outcomes including muscle strength, balance, and flexibility. The evidence identified was scarce regarding the interventions and outcomes selected, and the quality of the evidence was low–moderate. Although the studies included in the present review were RCTs, they presented high risk and unclear risk in aspects that affect the internal validity of the study and certainty in the measurement of the effect. These risks were related to the masking of randomized allocation^38,41^, the masking of outcome assessment^35,38,40^, and selective data reporting^39^. In addition, the sample sizes of the studies were small, which may explain the wide confidence intervals^34,35,37,39^. The loss to follow-up and the impact on statistical power could be related to the non-significant differences found in the equilibrium outcome^39^, which may have been the result of systematic type 2 error^42^.

No evidence was found for the outcome of flexibility. However, muscle stretching exercises were included as part of the training plan in two of the studies that examined muscle strength as the main outcome^37,41^. A previous study reported that the simultaneous training of flexibility and muscular strength improved muscular performance and the maintenance of improvements in muscular elongation^42^. Thus, flexibility may not have been included as a primary outcome in studies of the DS population because it was considered as a means to promote another outcome (such as muscle strength) and, in turn, as an indispensable element for effective and safe training^17,43^.

Another potential reason for the outcome of flexibility to not have been included as a therapeutic objective in previous studies is that children with DS exhibit characteristic muscle hypotonia, which is a decrease in the residual tension of the muscles at rest^44^. Hypotonia is in turn associated with decreased muscle strength, increased joint laxity, and increased flexibility in these children^45^. Therefore, improvement in flexibility may not be a primary objective of therapeutic exercise, particularly when there is controversy regarding whether increased flexibility directly promotes strength gains or, on the contrary, limits muscle strength gains^43^.

Regarding the parameters examined in the application of neuromuscular therapeutic exercise to improve muscle strength, the most common were frequency, intensity, and duration. The proposed therapeutic windows for the frequency of neuromuscular exercise in previous studies ranged from between two to three times per week, to 8 week^14,34^, to 24 weeks^15^. The approximate volume in this therapeutic window was three sets of 12 repetitions for each muscle group, the duration of each session varied between 35 and 75 min with an intensity between 60 and 80% of maximal voluntary contraction^14,34^.

Evidence regarding the use of neuromuscular exercise to improve balance reported the use of progressive muscle training 2–3 times per week in 45–60 min sessions for 10–24 weeks. These studies included isometric and isokinetic exercises and muscle stretching, as well as therapeutic vibration, exercise bikes and virtual reality with devices such as Nintendo Wii^34,35,37,38,40^.

The therapeutic window proposed in this review is related to the ACSM’s proposed guidelines for the evaluation and prescription of physical exercise^39^*.* The ACSM guidelines recommend a frequency greater than or equal to 2 or 3 days a week, a duration greater than or equal to 20 min, and an accumulated duration per week equal to or greater than 60 min, for the training of motor skills such as balance, coordination, gait, agility and proprioception in older adults and young people^17^.

The ACSM recommendations regarding prescription parameters are focused on physical exercise for potentially healthy adults^17^. There are no recommendations regarding parameters for the pediatric population because of the absence of supporting evidence in that age group. Future studies should evaluate the dose of neuromuscular exercise in potentially healthy children to define clear recommendations in this population. These can be extracted from the synthesis and analysis of scientific evidence or from the adaptation of existing scientific evidence regarding physical exercise in populations with specific conditions. Thus, future studies on therapeutic exercise should include the prescription parameters to get closer to a therapeutic dose of exercise in children with DS.

Despite the scarcity of evidence regarding the effects of neuromuscular exercise in the pediatric population with DS, an effect has been reported in a population of adults with DS. Sugimoto et al.^18^, reported significant changes in muscle strength, and functional task performance in adults and youth with DS when they performed neuromuscular exercise. To the best of our knowledge, the current study is the first systematic review to evaluate the effect of neuromuscular exercise in a pediatric population with DS from RCTs, contributing to the robustness of current evidence.

This systematic review synthesized neuromuscular exercise prescription parameters that have been shown to be effective, which is important for exercise-based rehabilitation care for children with DS. Future experimental studies should explore the effects of neuromuscular exercise in the pediatric population in early childhood (under 5 years) through play, given that the studies reported in the present review include the pediatric population from 8 years up. In children under 5 years, it is possible that the improvement of sensorimotor control allows to achieve fundamental motor patterns^46^ that appear late in children with DS compared with typically developing children^47^.

## Limitations of the study

A limitation of the study is the wide age range because multiple major developmental milestones occur in this span, and we found a low number of studies that met the eligibility criteria and that included children between 4 and 18 years. This made it difficult to carry out a comparative analysis between the prescription parameters and even the mode of application of the exercise in that life span. The low number of studies was also reported by the authors of the primary studies themselves.

## Conclusion

Despite the limited number of RCTs available in previous literature, neuromuscular exercise appears to be effective for the improvement of both lower limb and chest muscle strength in children over 8 years when isometric and isokinetic training is used as a mode of exercise in addition to other strategies, such treadmill training. There is also evidence for a positive effect on balance improvement trained with unstable surfaces and balance platforms, as well as strengthening of lower limbs. Future research is needed to investigate the effects of neuromuscular exercise in early childhood in more detail, as well as its effects on outcomes such as flexibility.

## Supplementary Information

Supplementary Information.
