# Supplementary material for: Neuromuscular exercise in children with Down Syndrome: a systematic review
Source: Sci Rep. 2022 Sep 2;12:14988. doi: 10.1038/s41598-022-19086-8 (PMC9440024; doi:10.1038/s41598-022-19086-8)
Supplement: Supplementary file 1 — Supplementary Information. [file 41598_2022_19086_MOESM1_ESM.docx]

Appendix 1. Meta-analysis by outcomes

1. **Muscular strength**
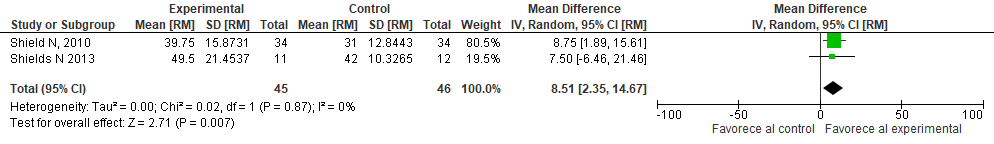


Figure a. Neuromuscular exercise with mechanotherapy equipment versus control, outcome: 1.1 Chest muscle strength (measuring instrument: [MR] maximal resistance).


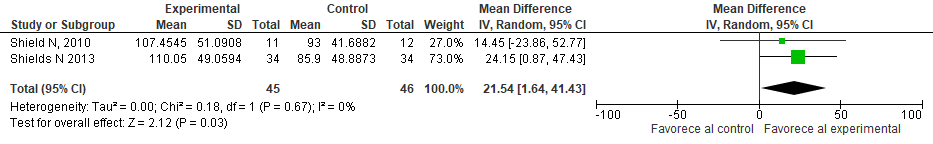


Figure b. Neuromuscular exercise with mechanotherapy equipment versus control. Outcome: 1.2 Leg muscle strength (measuring instrument: [MR] maximal endurance).
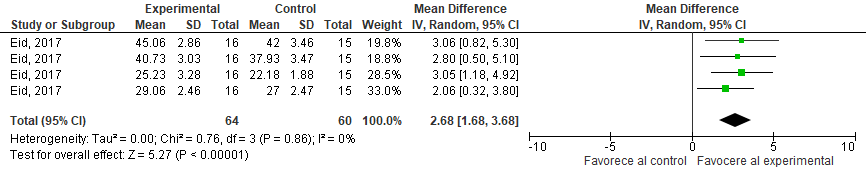


Figure c. Neuromuscular exercise with isokinetic training versus conventional physiotherapy outcome: 1.3 Lower limb muscle strength.


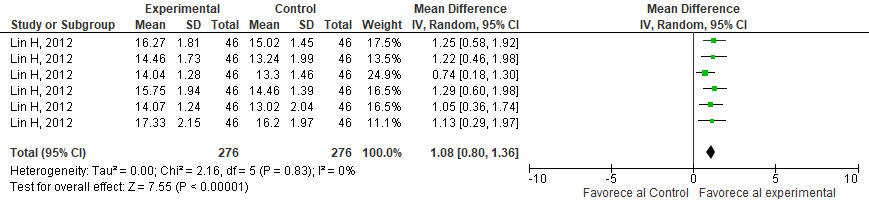


Figure d. Neuromuscular exercise using treadmill and Nintendo Wii versus control, outcome: 1.4 hip and knee muscle strength.


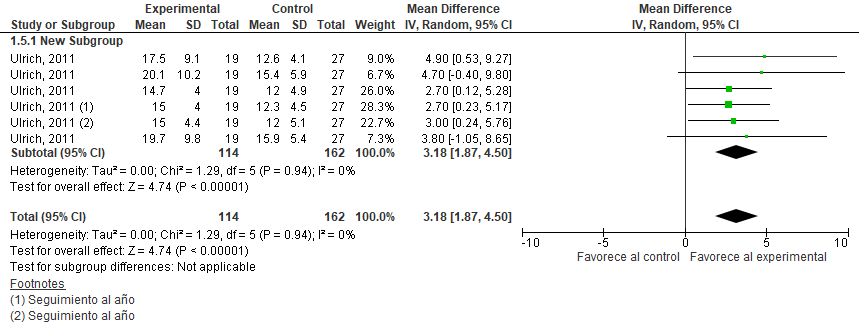
 Figure e. Neuromuscular exercise by isometric training versus control. Outcome: 1.5 Knee muscle strength.
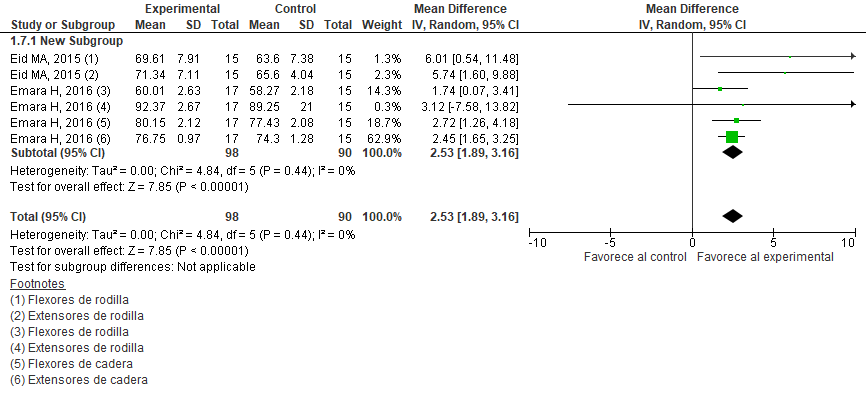
 Figure f. Neuromuscular exercise by therapeutic vibration plus conventional therapy versus conventional therapy. Outcome: 1.6. knee muscle strength.

**Balance**
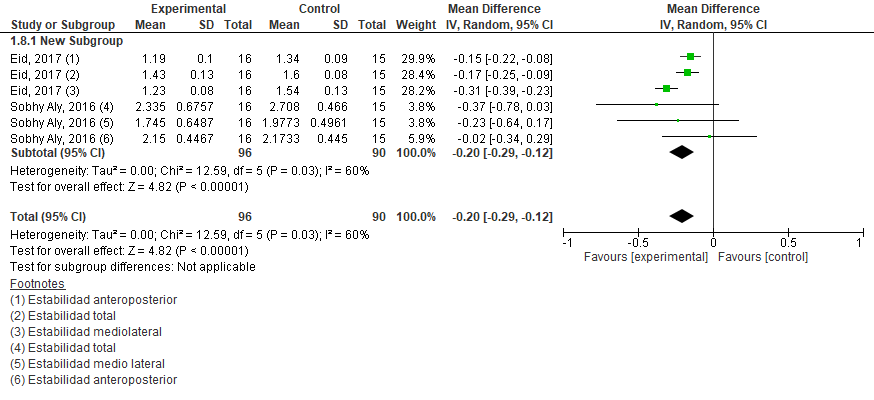
 Figure g. Neuromuscular exercise by conventional physiotherapy and isokinetic training/core stability exercises versus conventional therapy. Outcome: 2.1: Balance.


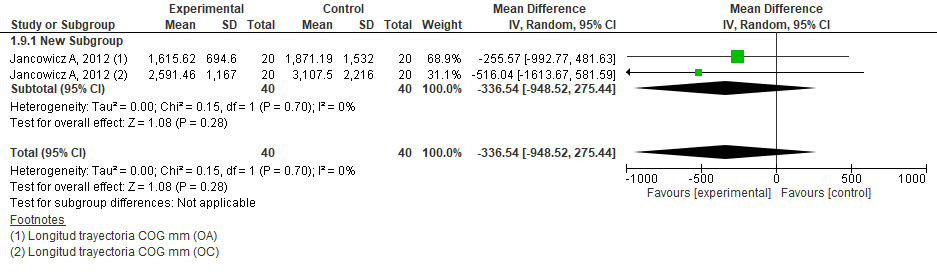


Figure h. Neuromuscular exercise using unstable surfaces and balls versus control. Outcome: 2.2. Balance.


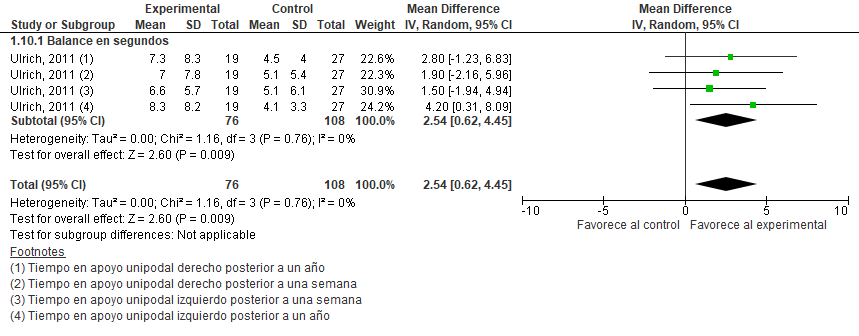


Figure i. Neuromuscular exercise through isometric training and unipodal balance versus control. Outcome: 2.3 Unipodal balance.
